# Group recognition in the German cockroach, a subsocial omnivore, is based on faecal odour preference that is modulated by coprophagy, diet and learning

**DOI:** 10.1098/rspb.2025.3042

**Published:** 2026-08-05

**Authors:** Ayako Wada-Katsumata, Jamora A. Hamilton, Madhavi L. Kakumanu, Coby Schal

**Affiliations:** Department of Entomology and Plant Pathology, North Carolina State University, Raleigh, NC 27695, USA

**Keywords:** German cockroach, coprophagy, aggregation, group recognition, olfactory learning, faeces, Behaviour

## Abstract

Faecal odours attract cockroaches to aggregations, where conspecific faeces provide nutrients and seed the gut microbiota. Because these odours arise from the gut and faecal microbial communities, which are shaped by coprophagy and diet, discrete aggregations within a heterogeneous landscape can produce distinct faecal odour profiles. How such odour diversity influences recognition, group affiliation and ultimately aggregation remains unclear. We manipulated coprophagy and then diet quality in gnotobiotic nymphs to generate groups with distinct faecal odour signatures, then raised them to the adult stage and collected their faeces for behavioural assays. Naive nymphs lacking coprophagy experience were equally attracted to faecal odours from all treatment groups, suggesting an innate attraction to conspecific faeces. By contrast, experienced nymphs preferred the faecal odour of their natal group over foreign groups, suggesting learned group-specific recognition. In associative learning assays pairing a faecal odour with a glucose reward, nymphs preferentially aggregated with the conditioned odour over novel odours. These results show that aggregation in neonate cockroaches is mediated by both an innate attraction to conspecific faecal odour and learned preferences for specific odourants encountered during coprophagy soon after they hatch. We propose that these mechanisms guide neonates towards natal aggregations, facilitating acquisition of a locally adapted gut microbial community.

## 1. Introduction

Semiochemicals—information-bearing chemicals—mediate chemical interaction between organisms [1]. For example, ‘emotive odours’ are those produced as a result of some transient physiological state or external stimulus [2]. Sex and alarm pheromones are included in this category. ‘Identifier odours’—distinctive odours of individual, sex, kin, nestmate, group and species—may be produced by a combination of factors, including the internal metabolism and hormonal state and the chemistry of scent glands, and by host-associated microorganisms [3]. These semiochemicals may be localized on the body surface or in urine, faeces or other secretions. Additionally, identifier odours may be generated de novo or accrued during foraging or resting in groups and act as aggregation pheromones [2]. Most pheromones are produced by the organism itself. However, the ‘fermentation hypothesis’ posits that symbiotic microbes inhabiting scent glands generate specific metabolites that serve as semiochemicals, which not only guide behaviour but also enable recognition of individuals and groups in mammals and other taxa [4-6]. Consistent with this hypothesis, various metabolites—and especially volatile organic compounds (VOCs) from various bacteria associated with skin, glands, digestive tract and faeces—also contribute to olfactory communication in many animal species [7,8].

Faeces-associated aggregation pheromones guide the behaviour of many social and gregarious species [9], including silverfish [10], bed bugs [11], earwigs [12] and cockroaches [13-15]. Although most studies have not explicitly assessed the origins of faecal aggregation pheromones, most of the identified VOCs appear to be gut bacteria metabolites and are not species-specific [16]. A natural extension of the fermentation hypothesis is that aggregation behaviour and group affiliation are guided by faecal semiochemicals. Previously we demonstrated that the absence of gut bacteria in gnotobiotic German cockroaches (*Blattella germanica*) dramatically reduced faecal volatile carboxylic acids (VCAs), which significantly reduced cockroach attraction to and aggregation in shelters [17]. Re-inoculation of their axenic guts with cultured faecal bacteria rescued the aggregation activity of their faecal extracts. Kakumanu *et al.* [18] revealed that 80–90% of the 16S rRNA reads were shared between the faeces and the respective gut microbiome in both laboratory-reared and field-collected cockroaches. Together, these findings suggest that faecal VCAs constitute an aggregation pheromone, and their production is strongly associated with gut microbiota; they highlight the pivotal role of gut bacteria in mediating insect–insect communication.

Although both the American (*Periplaneta americana*) and German cockroaches are generalist omnivorous feeders, the gut microbiome of the German cockroach, unlike that of the American cockroach [19], is highly dynamic, as it responds rapidly to different diets and reassembles in a diet-specific manner. For example, low- or no-protein diets support higher bacterial diversity than high-protein diets [20]. Other studies demonstrated that different diets reshape the gut microbiota and modulate olfactory sensitivity and preferences [21] but exert minimal effects on pathogen transmission [22]. Likewise, although German cockroaches from various field sites shared thousands of core bacterial taxa, we observed greater variation in the microbial community composition of cockroaches collected in apartments than in laboratory-reared cockroaches [18]. These observations support the idea that food and the local environment are major factors that shape the gut and faecal microbiomes, and they raise several related questions: How do cockroaches use variable diet-dependent gut microbial metabolites as a reliable aggregation pheromone? Do they conserve certain bacteria as core members of the gut microbiota, independent of diet, to produce the same faecal aggregation pheromone? Or do gut bacterial communities shaped by different diets produce variable, group-specific aggregation pheromone profiles and cockroaches tune their olfactory preferences to the faecal odour produced by their group members? Conceptually, the latter scenario would be similar to the use of variable, nest-specific signature mixtures or identifier odours (e.g. cuticular hydrocarbons (CHCs)) in nest and nestmate recognition in eusocial insects [23].

In this paper, we tested an extension of the fermentation hypothesis using the German cockroach as a model system. Newly hatched nymphs of this cockroach inoculate their gut microbiota through coprophagy on the faeces of groupmates, and the nymphal and adult microbiota is then shaped by their local environment and diet. Although we did not measure microbiota or odour profiles using analytical methods in this study, we previously demonstrated that aggregation pheromone production in this species is mediated by the gut microbiota [17]. Accordingly, we hypothesized that metabolites from the gut and faecal microbiota generate specific faeces-associated odour profiles that serve as group-specific variants and guide fidelity of group members to sheltering sites. To test this hypothesis, initially we demonstrated the influence of diet quality and coprophagy on nymphal development and faecal odour-guided aggregation behaviour. We used a two-stage experimental design. First, because the insect gut microbial community is shaped by habitat and diet [24], we tested whether different gut bacterial assemblages could be generated by inoculating gnotobiotic neonates with different faeces through coprophagy and maintaining the cockroach groups on different diets. Thus, the adults that emerged (parental generation) were expected to produce faeces with different odour profiles. In the second stage, we extracted the faeces of adult females (parental generation) and assayed the behavioural preferences of newly hatched nymphs (offspring generation) that were pre-exposed to various faecal odours.

## 2. Material and methods

### (a) Insects

The laboratory colony of *B. germanica* (Orlando Normal strain, also known as American Cyanamid, collected in a Florida apartment in 1947) used in these experiments was reared on rodent diet (Purina 5001 Rodent Diet, PMI Nutrition International, St Louis, MO, USA) and provided with glass-distilled water in cotton-stoppered vials. The main colony and all cockroaches used in this study were maintained at 27 ± 1°C, 40–70% relative humidity, and a 12 h L : 12 h D photoperiod.

### (b) Gnotobiotic first instar nymphs with axenic guts

The German cockroach forms an ootheca (egg case) that houses approximately 40–45 fertilized eggs. The female carries the ootheca for an approximately 20 day embryonic development. Embryos that are close to hatching develop pigmented eyes and a prominent green spot representing yolk in the hindgut. When a ‘green line’ was visible in the ootheca, the gravid female was briefly anaesthetized with CO_2_ and the ootheca was gently removed with forceps. We sterilized the exterior surface of oothecae by washing them for 1 min in a 0.5% bleach–water solution, then 70% ethanol in water for 1 min, and three times with water for 1 min each. All solutions were made with autoclaved water. To determine if the sterilization process was effective to generate axenic first instar nymphs (henceforth, first instars), two surface-sterilized oothecae were plated on tryptic soy agar (TSA) and plate count agar (PCA). After 3 days of incubation at 28°C, no colonies were visible in any of the treatments [25]. This surface sterilization procedure resulted in gnotobiotic neonates that lacked a gut microbiome, but they retained their maternally provisioned endosymbionts, namely *Blattabacterium*. These gnotobiotic nymphs could be maintained on TSA plates for several weeks without any bacteria growth (data not shown), confirming that their guts were axenic.

To generate the experimental groups (200 nymphs per treatment), 20–30 surface-sterilized oothecae were placed in a sterile 15 ml conical centrifuge tube (Falcon-Corning, Corning, NY, USA) and kept in a rearing cage (18.7 × 13.3 × 9.5 cm, T-79, Althor Products, Windsor Locks, CT, USA) that had been sterilized with bleach and 95% ethanol. To maintain moisture, sterilized glass test tubes containing autoclaved water were placed in the cage until the nymphs hatched. After hatching, the gnotobiotic first instars were randomly assigned to treatment groups.

### (c) Three types of diets

To obtain cockroach faeces of different qualities and odour profiles for the aggregation bioassays, we used three non-sterile diets with variable protein, carbohydrate, lipid and fibre contents: rodent diet was the standard diet for our colony and it contained moderate protein (24%), high carbohydrate (58%) and low lipids (5%) and fibre (5.2%); cat diet (Taste of the Wild Rocky Mountain Feline Recipe with roasted venison and smoked salmon, Schell & Kampeter, Meta, MO, USA) contained high protein (42%), low carbohydrate (21%), moderate lipids (18%) and low fibre (3%); rabbit diet (Oxbow Garden Select Adult Rabbit Food, Oxbow Enterprises, Murdock, NE, USA) had low protein (12%), high carbohydrate (43%), low lipids (2.5%) and high fibre (22–26%). Each diet was crushed in a blender and placed into separate Ziploc bags. Sterilized rodent diet was prepared using gamma radiation at a dose of 10 000 Gy at the USDA-APHIS-PPQ S&T Otis Laboratory (Buzzards Bay, MA, USA). To verify the sterilization procedure, two samples of the irradiated diet were plated on TSA and PCA media using four plates per sample. After 3 days of incubation at 28°C, no colonies or visible growth were detected in any of the diet and control plates.

### (d) Generating three treatment groups

To test the effects of bacteria sources and diet on aggregation preferences to faecal extracts, we generated three experimental groups as detailed in electronic supplementary material, table S1. General procedures common to all three treatment groups were as follows. Gnotobiotic first instars (with axenic guts) were placed in a sterile rearing cage (100 nymphs per cage). Each cage contained an autoclaved egg carton shelter and autoclaved water in a sterilized glass test tube (25 × 150 mm, Fisherbrand, Fisher Scientific Company, Hanover Park, IL, USA). Each of the three experimental groups had two replicate cages. Routine maintenance every two weeks was conducted within a biosafety cabinet. The nymphs and their shelter were transferred without anaesthesia to a new rearing cage to maintain a clean, sterile environment. Water and diets were replaced as needed. The first date that nymphs moulted to the next development stage in each cage was recorded. The date of each adult emergence, the sex and body mass were recorded daily.

To obtain faecal materials and first instars for bioassays, newly emerged adult males and females from each treatment group were transferred daily into sterile mating cages with sterile shelter, diet and water. Gravid females with a ‘green line’ visible in their oothecae were then transferred to a sterile hatching cage with sterile shelter and water. Newly hatched gnotobiotic nymphs were used in bioassays. Thus, the adults were considered the end of the parental generation, and the offspring nymphs they produced were used in bioassays.

To collect fresh faeces from adult females, 20–50 females carrying oothecae were collected. We carefully dislodged their oothecae, which stimulated the females to initiate a new gonotrophic cycle of feeding, oocyte provisioning and faeces excretion. The females, along with a sterile shelter, diet and water were placed in a sterile cage with a screen (12 Mesh T316 Stainless, 0.58 mm wire diameter, 1.53 mm opening, TWP, Berkeley, CA, USA) at the bottom. We collected the faeces that passed through the screen during one week. The diet was placed in a small Petri dish to prevent it from falling through the screen, but any diet debris was carefully removed from the faeces under a microscope.

The three treatment groups were defined as follows (electronic supplementary material, table S1):

#### Different-diets (no coprophagy).

To evaluate the effect of diet on the development of gnotobiotic nymphs, 1-day-old first instars with axenic guts received sterile water and one of three non-sterile diets (1 g of rodent, cat or rabbit diet, hereafter denoted D-Rodent, D-Cat and D-Rabbit, with ‘D’ representing ‘Diet’) in a Petri dish (60 mm diameter, Falcon-Corning, Glendale, AZ, USA); they were not inoculated with faecal bacteria (no coprophagy). We expected their faecal odours to differentiate as their gut microbiomes diverged on the different diets and associated environmental bacteria during nymphal development.

#### Coprophagy & different-diets.

To evaluate the effect of coprophagy on the development of gnotobiotic nymphs, 1-day-old neonates with axenic guts received sterile water and non-sterile faeces (0.5 g on a 60 mm Petri dish) excreted by virgin females from the laboratory colony; these females were maintained on non-sterile rodent diet. The nymphs were given 3 days to inoculate their guts with faecal bacteria. Then, the Petri dishes with faeces were removed and groups of nymphs received one of three different non-sterile diets as in the ‘D’ treatment above (hereafter denoted FD-Rodent, FD-Cat and FD-Rabbit, with ‘F’ representing ‘Faeces’). We expected the ingested faecal bacteria to inoculate all nymphs with a common gut microbiota, which would then be differentiated by the different diets and environmental bacteria during nymphal development, producing unique faecal odour profiles.

#### Coprophagy & sterilized-rodent-diet.

We expected cockroach groups raised on different diets to differ in their gut and faecal microbiomes and therefore in their faecal odours. To test whether inoculation with different faecal bacteria via coprophagy would impact the development and olfactory preferences of nymphs fed the same diet, 1-day-old gnotobiotic neonates received sterile water and three different types of faeces (from females in the FD-Rodent, FD-Cat or FD-Rabbit groups) for 3 days (0.5 g on a 60 mm Petri dish). After removing the Petri dishes with faecal inoculum, the nymphs were offered irradiated rodent diet. Hereafter, these treatments are denoted RoFD-stRodent, CaFD-stRodent and RaFD-stRodent, with RoF, CaF and RaF representing rodent, cat and rabbit faeces, respectively, and stRodent representing sterilized rodent diet. Previous studies showed that diet quality strongly influenced and shaped gut bacterial communities [20-22]. Therefore, we expected that gnotobiotic neonates inoculated with different bacteria from different faecal sources would develop divergent gut microbiota. At the same time, we expected that the common sterile diet throughout nymphal development and during the adult stage would converge digestion pathways and the gut environment and thus unify the gut bacterial communities of the three treatments.

We also set up a single cage containing 100 nymphs to monitor development of gnotobiotic nymphs after a brief starvation period that mimicked the exposure to faeces, followed by exposure to sterilized diet throughout nymphal development. These gnotobiotic neonates did not receive faeces for 1 day (starved, no coprophagy) and then received irradiated rodent diet (No-coprophagy & sterilized-rodent-diet).

### (e) Bioassays using first instars

Faeces were collected from three groups of adult females fed the three non-sterile diets (i.e. rodent, cat or rabbit diets). The faeces were weighed and homogenized (50 mg faeces ml^−1^ sterile water) in a 1.5 ml Eppendorf tube. We vortexed the suspension for 3 min, then centrifuged (20 817 g for 1 min at 4°C) and collected the supernatant in a 1.5 ml sterile Eppendorf tube. We defined the concentration of this initial extract as ‘1×’ (50 μg faeces-equivalents μl^−1^).

1. Dose–response assays. Faeces originating from the three different treatments varied in their effectiveness at stimulating aggregation. To standardize the stimuli used in two-choice aggregation assays, we estimated the effective concentration (EC) of each faecal extract using first instars that hatched from the respective diet treatment (i.e. offspring nymphs). Each of the faecal extracts from adult females fed different diets was diluted 10-fold (0.1× = 5 μg faeces-equivalents μl^−1^) and 100-fold (0.01× = 0.5 μg faeces-equivalents μl^−1^) with sterile water. Two accordion-style folded shelters (20 × 6.7 mm, Whatman no. 1, Pittsburgh, PA, USA) were placed at opposite edges of a Petri dish (60 mm). One shelter was treated with 1 μl of the faecal extract of the tested nymph’s own treatment group. The other shelter was treated as control with 1 μl of sterile water. A single first instar was allowed to walk onto an autoclaved wooden stick (140 × 5 mm, Eisco^™^ Wooden Splints, Fisherbrand) and guided to descend onto the centre of the arena; thus no anaesthesia was used. Bioassays were initiated during the photophase, when cockroaches normally aggregate, and the position of the nymph within the arena was recorded 2 h later. We conducted 20 to 60 assays per concentration. The EC_50_ (effective concentration) and EC_70_ were calculated from the resulting dose–response curve for each group (electronic supplementary material, figure S3). Based on these results, we used 3 μl of 5 μg faeces-equivalents μl^−1^ faecal extract for all subsequent bioassays.
2. Two-choice aggregation assays. The experimental aggregation assays were identical to the dose–response assays, except we used 100 × 15 mm Petri dish arenas (Fisherbrand) and tent-shaped shelters (20 × 20 mm, Whatman no. 1). Based on the dose–response bioassays, shelter 1 and shelter 2 were treated with 3 μl of sterile water or faecal extract (5 μg faeces-equivalents μl^−1^). The overall response of nymphs was calculated as response (%) = 100 × (no. nymphs choosing shelter 1 and shelter 2/total no. tested nymphs). A preference index was calculated as preference (%) = 100 × (no. nymphs choosing either shelter 1 or shelter 2/total no. nymphs that chose shelter 1 and shelter 2).

### (f) Olfactory associative learning

Using gnotobiotic neonates, obtained by surface-sterilizing the oothecae of lab-colony females fed rodent diet, we generated five experimental groups. Newly hatched groups of approximately 200 gnotobiotic neonates with axenic guts were placed on a sterile filter paper strip (20 × 80 mm, Whatman no. 1) that served as a shelter in a sterile 50 ml conical centrifuge tube (Falcon-Corning, Corning, NY, USA) with sterile water in a 1.5 ml Eppendorf tube.

1. No-conditioning (N). Nymphs did not receive any conditioning.
2. Coprophagy-conditioning (F) (faecal nutrient and faecal odours). To mimic natural coprophagy, nymphs received sterile water in a 1.5 ml Eppendorf tube and 0.2 g of non-sterile faeces collected from females fed non-sterile rodent diet (FD-Rodent) moistened with 10 μl sterile water. In this paradigm, nymphs engaged in coprophagy while being exposed to faecal odour for either 1 day or 3 days.
3. Glucose–agar-conditioning (Glu) (glucose nutrient only). Nymphs received 2 ml of a blue-coloured glucose–agar mix (1 mol l^−1^ glucose, 1% agar and 0.5 mmol l^−1^ erioglaucine). Erioglaucine, also known as Brilliant blue FCF, is a food-grade dye that is not toxic to cockroaches [26]; it was used to confirm that the nymphs consumed the glucose–agar mix. Nymphs were exposed to the glucose–agar for either 1 day or 3 days but not to faecal odour other than their own faeces.
4. Faecal odour-conditioning (FO) (faecal odour only). Nymphs received faecal odour from 0.2 g of FD-Rodent female faeces moistened with 10 μl sterile water. Faeces were placed in a 1.5 ml Eppendorf tube capped with a piece of cheesecloth (Monarch, Knoxville, TN, USA). Thus, nymphs were exposed to faecal odour but prevented from contacting the faeces for either 1 day or 3 days.
5. Glucose–agar & faecal odour-conditioning (Glu+FO). Nymphs received 2 ml of blue-coloured glucose–agar mix, as above, while being exposed to faecal odour, as above. Thus, nymphs were prevented from contacting the faeces for either 1 day or 3 days but were exposed to faecal odour while eating glucose–agar.

Faecal odour preferences of conditioned 1-day-old nymphs (1 day conditioning) and 3-day-old nymphs (3 days conditioning) were tested in two-choice aggregation assays using the faecal extracts of FD-Rodent, FD-Cat and FD-Rabbit (3 μl per shelter, 5 μg faeces-equivalents μl^−1^). We expected that nymphs in Experiment 1 (no conditioning) would be equally attracted to different faecal extracts, while nymphs in Experiment 2 (coprophagy-conditioning) would prefer the FD-Rodent faecal odours over other faecal odours (FD-Cat and FD-Rabbit). If this faecal odour preference was due to associative olfactory learning during feeding, nymphs in experiments 3 (glucose nutrient only) and 4 (faecal odour only) should not have a specific preference for FD-Rodent faecal odour. On the other hand, in Experiment 5 (glucose–agar and faecal odour-conditioning) nymphs were expected to prefer FD-Rodent faecal odours. The preference index was calculated using 30 replications of each assay.

### (g) Data analysis

We found no differences in the nymphal development periods between the two replicates within each treatment group (electronic supplementary material, figure S1). Therefore, we combined both replicates for the analysis of nymphal development times (adult emergence) (electronic supplementary material, figure S2) and the bioassays that followed. The effects of diet and coprophagy on nymphal development and adult body mass (parental generation) of the Different-diets group and Coprophagy & different-diets group were tested by two-way ANOVA (*α* = 0.05) followed by Tukey’s honestly significant difference (HSD), which enabled multiple comparisons of main (fixed) effects, including development with versus without coprophagy (coprophagy main effect) across the three diets (diet effect) and the interaction of these two factors (coprophagy * diet). Odour preferences of first instars (offspring generation) were assessed using a *χ*^2^-test of independence (*α* = 0.05). All statistical analyses were performed in JMP (Student edition 18, Cary, NC, USA). Details of all statistical tests are shown in the electronic supplementary material.

## 3. Results

### (a) Diet and coprophagy impact nymphal development and adult body mass

**Figure 1.**
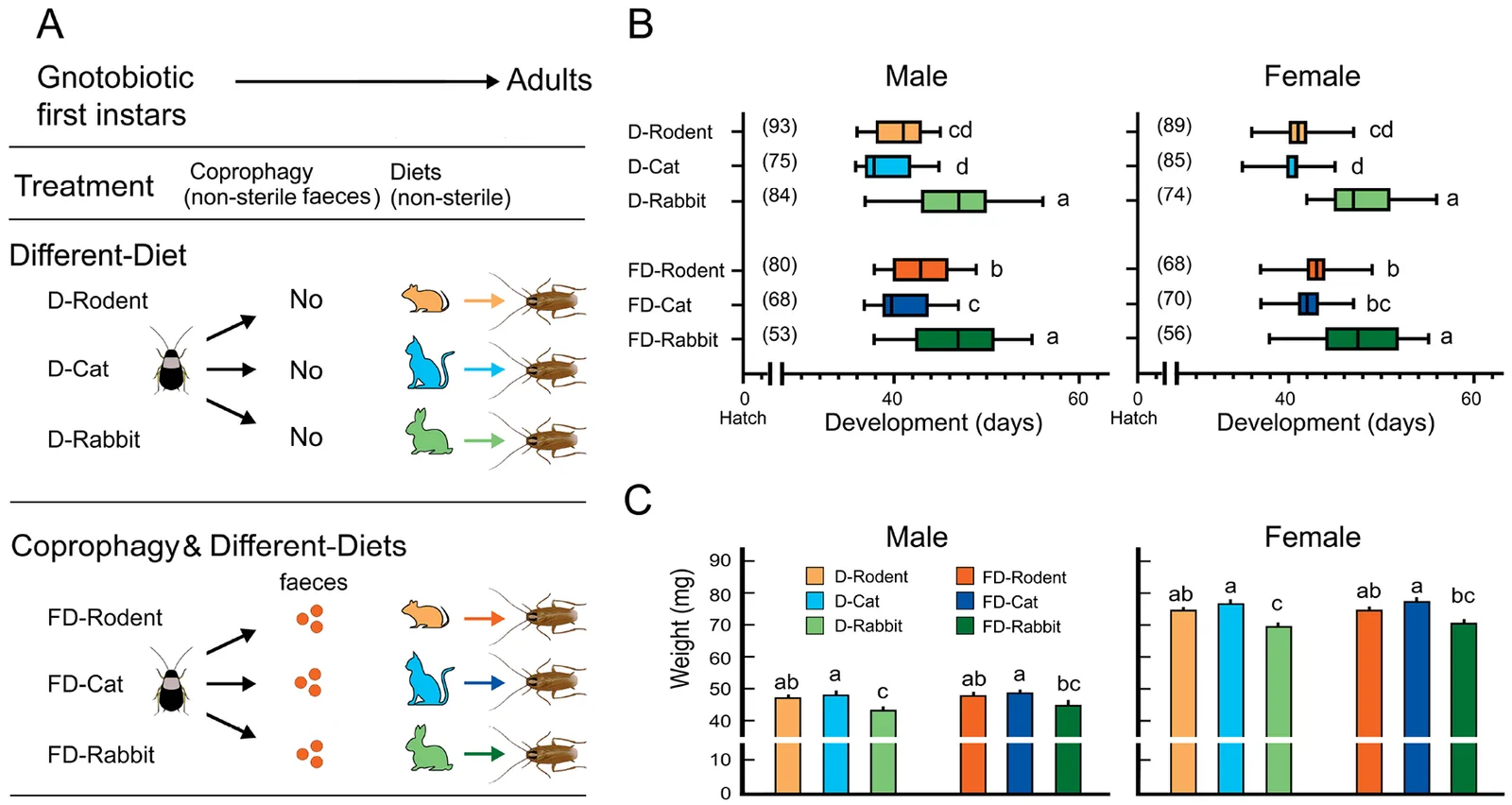
Effects of diet and coprophagy on nymphal development and adult body mass. (A) Experimental design in which gnotobiotic first instars with axenic guts (black and grey) received three different non-sterile diets with or without coprophagy (details are shown in electronic supplementary material, table S1). (B) Nymphal development duration, which corresponds to days to adult emergence separately for males and females, and (C) adult body mass of males and females. Each treatment group consisted of two replicates, each with 100 nymphs, for a total of 200 nymphs per treatment. The respective number of males and females (*n*) for each treatment is shown in parentheses, and different lower-case letters indicate significant differences in two-way ANOVA followed by Tukey’s HSD (*p* < 0.05) (electronic supplementary material, table S2).

Different diet treatments resulted in different nymphal development rates and adult body mass (figure 1). Cat diet tended to support faster nymphal development (figure 1B; electronic supplementary material, figure S1) and a narrower adult emergence period in both females and males than our standard rodent diet (electronic supplementary material, figure S2); however, body mass of adult females and males were not significantly different between the two diets (figure 1C). Rabbit diet caused delays in nymphal development (figure 1B; electronic supplementary material, figure S1) and adult emergence ranged over a wider period (electronic supplementary material, figure S2). Adult body mass was significantly lower on rabbit diet than on rodent or cat diets (figure 1C).

Nymphs in the Different-diets group tended to develop faster than nymphs in the Coprophagy & different-diets group and mortality in the Different-diets group tended to be lower than in the Coprophagy & different-diets group (electronic supplementary material, figure S1). These differences were likely because the Coprophagy & different-diets nymphs experienced 3 days of feeding solely on faeces before they were offered their respective diets. Nevertheless, there were no differences in adult body mass between these groups (figure 1C). Overall, these results indicated that rabbit diet was nutritionally suboptimal for German cockroach nymphs and that inoculation of the gut with faecal bacteria through coprophagy did not overcome the effect of poor diet quality during nymphal development. Cat diet was more suitable than rabbit diet for development and adult body mass, but coprophagy (i.e. presence of a gut microbiota) did not affect these patterns.

### (b) Diet and coprophagy impact faecal odours that guide aggregation preferences

**Figure 2.**
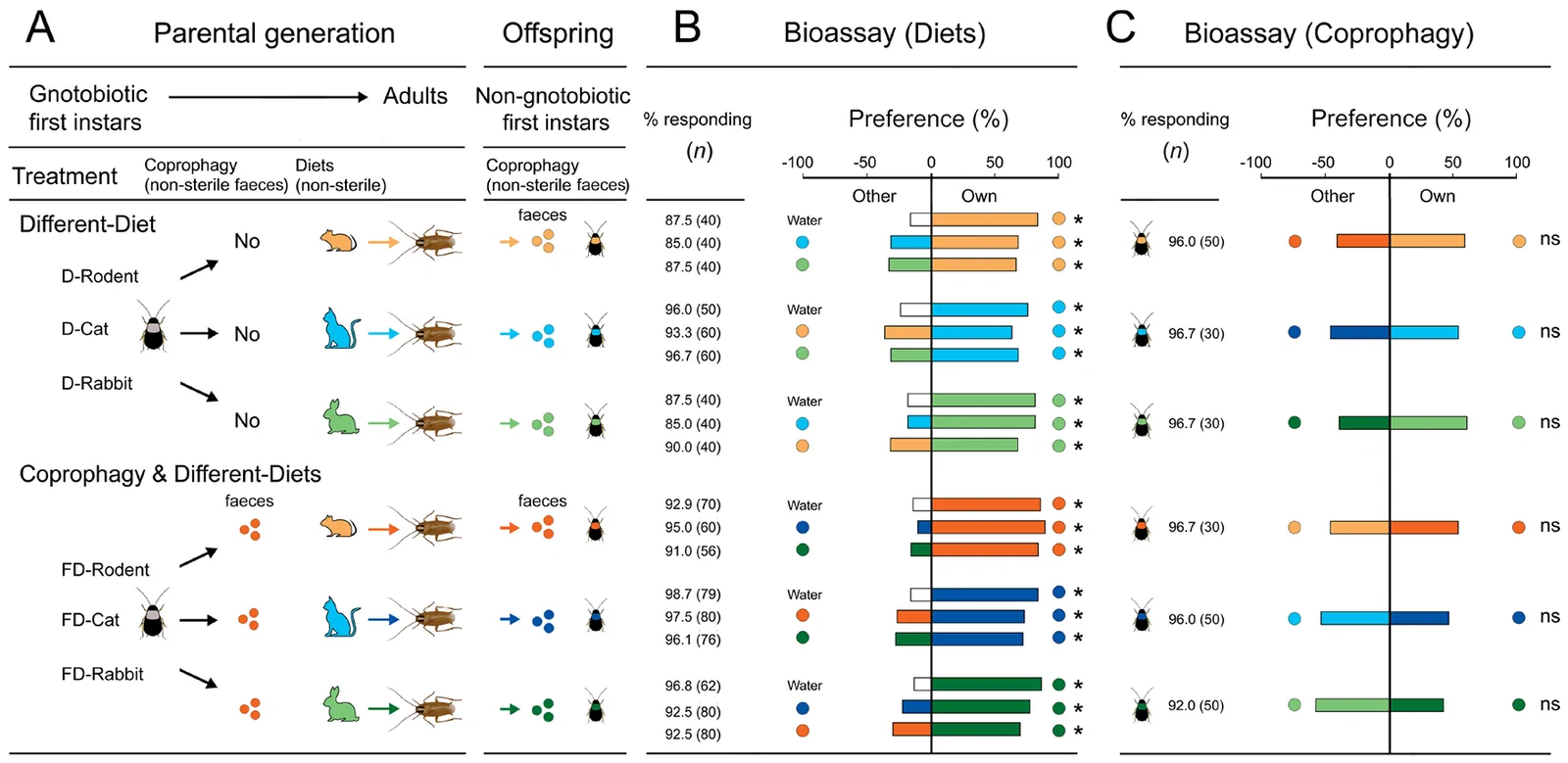
Effects of coprophagy (faecal inoculation of the gut microbiome) followed by different diets on faeces odours of the parental generation as assessed by faecal odour preferences of first instar offspring. (A) Experimental design showing gnotobiotic neonates (black and grey) provided with a common faecal inoculum through coprophagy followed by different non-sterile diets throughout nymphal and adult development of the parental generation. In the Different-diets treatment gnotobiotic neonates were not provided faeces (no coprophagy) and given different non-sterile diets. In the Coprophagy & different-diets treatment gnotobiotic neonates were provided a common faecal source (coprophagy on the same faeces) followed by different non-sterile diets. The faeces generated from these treatments were used in two-choice preference assays of first instars that hatched in each treatment group (offspring generation, details are shown in electronic supplementary material, table S1). (B,C) Odour preferences of conventional (non-gnotobiotic) nymphs given a choice between shelters treated with faecal extracts from their own parental treatment group (Own) or from a different treatment group (Other). Different-coloured circles indicate faecal extracts from different treatment groups and different-coloured bars represent percentage of nymphs that chose the same-coloured faecal extracts. The numbers of replicate bioassays (*n*) are shown in parentheses, and asterisks indicate a significant preference for a faecal extract (*χ*^2^-test, *p* < 0.05) (electronic supplementary material, tables S3 and S4).

To test whether faecal odours from different sources differentially attract nymphs, we conducted two-choice aggregation assays using first instars exposed to faeces extracts from the parental group in the Different-diets and Coprophagy & different-diets groups (figure 2A). Nymphs preferred the odour extracted from the faeces of their own parental diet treatment over other faecal odours (figure 2B), suggesting that diet quality impacted the odourant profile of the faecal aggregation pheromone and facilitated group recognition. However, in binary choice assays, nymphs did not exhibit any preferences among the three faecal odours that differed only in the presence or absence of coprophagy in the parental generation (figure 2C). These findings suggest that the faecal metabolites of the parental generation adults were shaped by diet quality throughout nymphal development rather than the brief inoculation through coprophagy early in their first nymphal stage.

**Figure 3.**
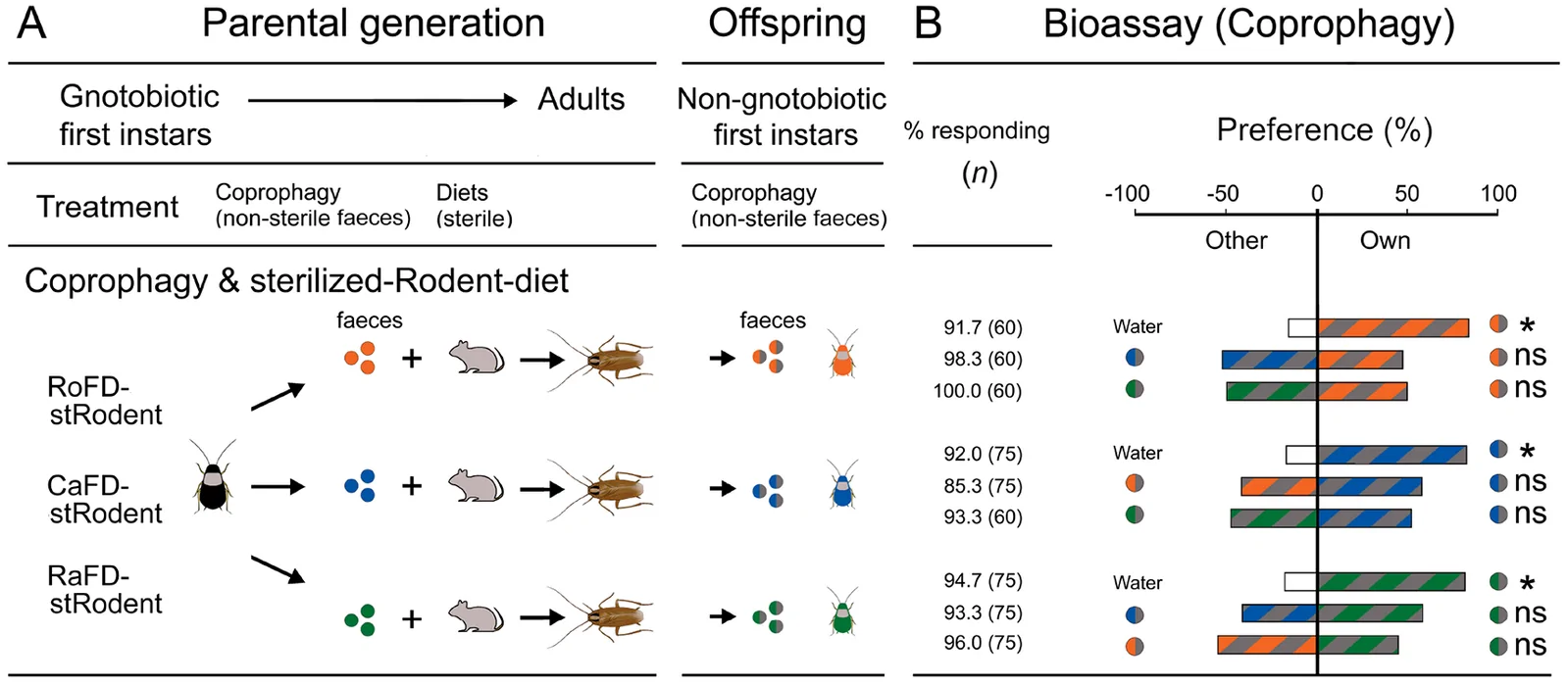
The effects of early divergence of the gut and faecal microbiomes through differential coprophagy (Coprophagy & sterilized-rodent-diet) on faecal odour preferences. (A) Parental generation gnotobiotic neonates with axenic guts (black and grey) were inoculated through coprophagy with different non-sterile faeces and then raised on a common sterilized rodent diet (stRodent). Faecal odour preferences of first instars that hatched in each treatment group (offspring generation, details are shown in electronic supplementary material, table S1) were tested in two-choice aggregation assays using faecal extracts obtained from adult females in the parental generation. (B) Faecal odour preferences of conventional (non-gnotobiotic) nymphs given a choice between faecal odours from their own parental treatment (Own) or faecal odours from a different treatment (Other). Different-coloured circles indicate faecal extracts from different treatment groups and different-coloured bars represent percentage of nymphs that chose same-coloured faecal extracts. The numbers of replicate bioassays (*n*) are shown in parentheses. Asterisks indicate a significant preference for a faecal extract (*χ*^2^-test, *p* < 0.05) and ns indicates no significant preference (*p* > 0.05) (electronic supplementary material, table S5).

To confirm the effect of coprophagy on nymphal development and the relative effectiveness of various faecal odours in guiding aggregation, we allowed gnotobiotic neonates to engage in coprophagy on different faecal samples and then feed on a common sterilized rodent diet (Coprophagy & sterilized-rodent-diet). Gnotobiotic neonates from the laboratory colony were provided faeces from adults emerging from the three Coprophagy & different-diets groups, and all nymphs were raised on the same sterile rodent diet (stRodent) until adults emerged. There were no significant differences in the development of nymphs in the RoFD-stRodent, CaFD-stRodent and RaFD-stRodent treatments (electronic supplementary material, figure S1). In two-choice aggregation assays, offspring generation nymphs preferred the odour of their natal faecal extract over no odour (water). However, nymphs did not discriminate their own group’s faecal odour from odours derived from related experimental groups that were inoculated by different faeces but fed on the same diet (figure 3). These results indicate the possibility that, although the guts of the parental generation nymphs were inoculated with different microbial communities, exposure to the same sterile diet throughout nymphal development and during the adult stage likely caused the faecal odours to converge, precluding nymphs from discriminating their own from other colonies’ faecal odours.

### (c) Plasticity of odour preference of first instars

**Figure 4.**
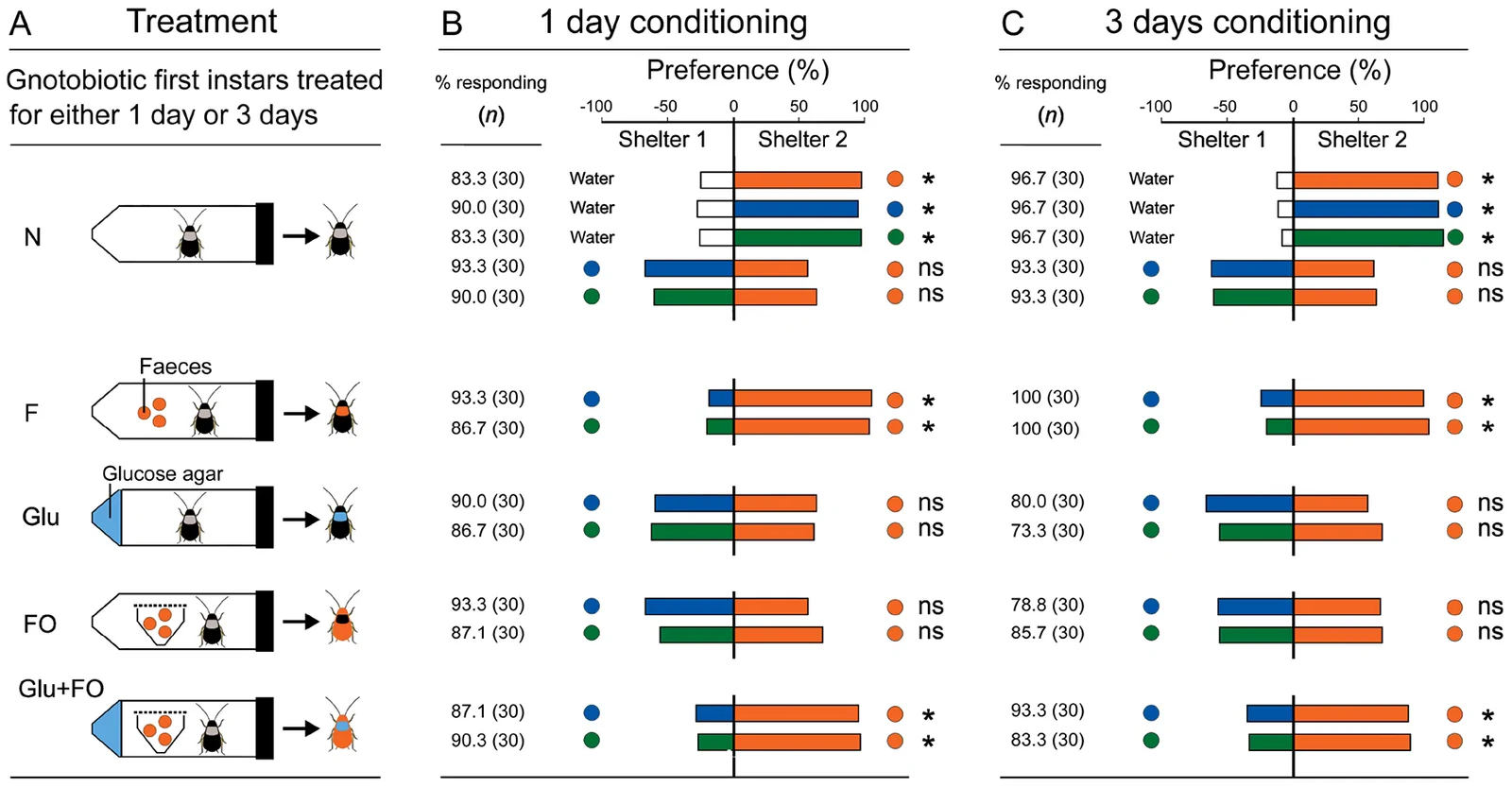
Olfactory associative learning guides faecal odour preference of neonates. (A) Experimental design of conditioned and unconditioned nymphs. Gnotobiotic neonates (black and grey) received glucose only (blue base of the conical tube; unconditioned nymphs) without conditioning (*n*). Other axenic neonates were conditioned with coprophagy (faecal olfaction and gustation) on the faeces of the FD-Rodent parental generation (F), glucose–agar only (Glu), faecal odour of FD-Rodent faeces only (FO) or a combination of glucose and the faecal odour of FD-Rodent faeces (Glu+FO). Faecal extracts were obtained from the Coprophagy & different-diets groups (details are shown in electronic supplementary material, table S1). (B,C) Faecal odour preferences of 1-day-old nymphs (1 day conditioning) and 3-day-old nymphs (3 days conditioning) from (A) given a choice between various faecal extracts. Different-coloured circles and bars represent different treatment groups as in figure 3. The number of replicate bioassays (*n*) are shown in parentheses. Asterisks indicate a significant preference for a faecal extract (*χ*^2^-test, *p* < 0.05) and ns indicates no significant preference (*p* > 0.05) (electronic supplementary material, table S6).

To test whether faecal odour preference of first instars is mediated by olfactory associative learning, we coupled faecal odours with glucose, the unconditioned nutrient reward stimulus (figure 4; electronic supplementary material, table S6). First, we kept gnotobiotic neonates (no prior exposure to any faecal odours) for either 1 day or 3 days without faecal odour and no food (N). These naive nymphs were attracted to all three faecal odours generated from the FD-Rodent, FD-Cat and FD-Rabbit groups, indicating that faecal extracts contain VOCs that attract conspecific nymphs. However, the naive nymphs did not discriminate the FD-Rodent faecal extract from the other two faecal odours. Second, we exposed nymphs to FD-Rodent faeces without glucose–agar (F), allowing them to experience odours and obtain faecal nutrients during coprophagy [27]. These nymphs preferred FD-Rodent faecal odour over the other two faecal odours, showing that early experience with a faecal source conditioned their subsequent preferences. In the third (FO) and fourth (Glu) experiments, we demonstrated that faecal odour only did not induce odour preference to FD-Rodent faecal odour. In the fifth experiment, we offered nymphs a glucose–agar mix while they were exposed to faecal odour (from the FD-Rodent treatment group) but without contact with the faeces (Glu+FO). These odour-conditioned nymphs preferred the FD-Rodent faecal odour over the other two faecal odours. These results imply that nymphs have an innate odour preference for a range of conspecific faecal odours, but when they experience a specific faecal odour with a food source, they learn that faecal odour variant and can subsequently discriminate it from other odour variants.

## 4. Discussion

### (a) Faecal odours guide aggregation behaviour

In a previous study [17], we demonstrated that faecal VCAs mediate both attraction and aggregation of nymphs in treated shelters. Moreover, we showed that odours emanating from faecal bacterial isolates promote aggregation behaviour. Based on characterization and manipulation of the gut microbiome of *B. germanica* [20-22,28-32], we hypothesized that exposure of nine experimental groups to differential coprophagy and different diets would generate divergent gut microbial communities that would emit an array of different faecal compounds, including VCAs.

Bioassays using faecal extracts from different treatments revealed two important characteristics of faecal odour discrimination by nymphs. First, their faecal odour preferences are plastic, but they prefer to aggregate with odours consistent with their own early exposure to faecal odours. Second, faecal odour preferences of nymphs are not driven by the coprophagy history and diet quality of their parents. For example, although rabbit diet was suboptimal, resulting in slower nymphal development and delayed emergence of adults, their offspring chose the adult (natal) faecal odour over faecal odours from the more developmentally optimal rodent and cat diets, suggesting that the aggregation behaviour is not governed by an evaluation of faecal nutrient quality.

Ishii [33] demonstrated that German cockroach nymphs aggregated on faeces-conditioned filter papers with not only conspecific odour but also the odours of other species. Similar broad acceptance of heterospecific faecal odours has been reported across multiple cockroach species [34,35]. Paradoxically, other researchers found not only that some species can discriminate conspecific from heterospecific faecal odours [13], but also significant odour-based strain and group preferences in the German cockroach [36,37]. The mechanisms that underlie acceptance of the odours of other species and at the same time intraspecific group recognition are unclear.

In this study, we demonstrated that cockroach preferences are highly plastic and guided by faecal odours that appear to reflect different gut microbial communities. In a related study of aggregation in *B. germanica*, we noted that two cockroach colonies on separate continents emitted different faecal odour blends, but with some shared odourants [9,38]. Consistent with these and the present results, we propose that divergent gut and faecal microbiomes emit divergent yet overlapping odour profiles, which would explain not only group recognition but also inter-species preferences, depending on prior experience of nymphs and the bioassay design. Likewise, these overlapping odours might be responsible for the innate faecal odour preference that we observed in naive nymphs with no prior experience with faecal odour (figure 4, N treatment). It will be fascinating to know which compounds stimulate the innate faecal odour preference and if they originate from a common species-specific source, such as rectal pads, which were suggested by Ishii & Kuwahara [15] as the source of aggregation pheromone, or if they originate from shared members of the gut microbiota that emit common compounds.

### (b) Mechanism and proposed model for aggregation in cockroaches

Olfactory associative learning experiments revealed that naive nymphs deprived of experience with both faecal odour and glucose (N) were attracted to each of the three different faecal odours with no apparent preferences. By contrast, nymphs that received glucose while exposed to a specific faecal odour preferred the odour that they experienced over other faecal odours (figure 4). These results indicate that olfactory learning supports discrimination among faecal odours. As neonate nymphs hatch with a microbe-free gut, coprophagy within the aggregation provides them with their first meal of groupmate-provisioned faecal nutrients (analogous to glucose in our experiment) coupled with a group-specific faecal odour. This experience shapes the preference of first instars through olfactory associative learning and memory, enabling them to recognize their group’s aggregation site. Cockroaches, including *B. germanica*, have served as excellent models for learning and memory studies [39-42], but future research should investigate whether first instars have comparable learning ability to older nymphs and adults.

Our results suggest that gut and faecal microbes shaped by coprophagy and diets mediate local group-specific faecal odours that guide group recognition in newly hatched nymphs. These behavioural studies provide compelling empirical evidence for the fermentation hypothesis, but detailed microbiome and faecal odour analyses are needed to reinforce this model. Based on the behavioural results presented here, we propose that the aggregation pheromone of the German cockroach consists of two general classes of faecal odours: odours that stimulate innate odour preferences, and group-specific faecal odours that represent local variants guiding group affiliation. There is a possibility that aggregation behaviour is mediated not by entirely different classes of compounds, but rather by differences in their relative ratios or by other factors, including life-history-dependent exposure windows that shape learning. The chemical profile of each local odour variant may represent the genetic background of the cockroach population and environmental factors such as food quality, ingested microbes and environmental chemicals that structure the microbial community. In turn, locally selected gut and faecal bacterial assemblages associated with metabolic pathways for nutrient digestions [20-22] and insecticide resistance [43-45] may endow faeces with unique odours.

Wyatt [1] distinguished between ‘pheromone’ (a blend that elicits an unlearned response from a receiver) and an ‘odour signature’ or ‘signature mixture’ (an odour or odour blend that requires prior learning to elicit a response). For example, in nest and nestmate recognition of social insects, extrinsic (environmental) and intrinsic (physiology, genetics) factors shape unique nest odours—usually a mix of CHCs—as signature mixtures [46]. The species-specific signature is plastic, as workers must learn to recognize their nest’s unique chemical profile so they can distinguish nestmates from foreign invaders.

Aggregation towards faeces in the German cockroach appears to be mediated by group recognition using locally specific faecal odours as signature mixtures, which might be functionally similar to the system of group or nest recognition in social insects to enhance various benefits of group-living. This model clarifies why nymphs from our colony were attracted to a mix of VCAs based on this colony’s faecal odour [17], whereas nymphs from another lab colony were attracted to a different mix of VCAs [38]. It might also explain the variable chemistry of putative aggregation pheromone components identified in previous studies of the German cockroach.

### (c) Effects of coprophagy (gut microbiome inoculation) and diet on nymphal development

The biology of the German cockroach is supported by a dual symbiont system [47]. The endosymbiont *Blattabacterium* is distributed throughout the fat body and is vertically transmitted through the oocytes. It serves a vital collaborative function in nitrogen recycling with its host [28,48,49]. In our study, cockroaches in all treatments retained *Blattabacterium*. The second symbiont system is a diverse and highly abundant gut microbiota, which is horizontally transmitted mainly through coprophagy, a common behaviour in various animal species [50-57]. After its inoculation, the gut microbial community is shaped by the physiological state of the host, diet and environmental factors including pathogens (production of antimicrobial agents), antibiotics (expression of antibiotic resistance genes), pesticides (upregulation of resistance mechanisms) and other biotic and abiotic factors that may contribute to adaptation of the microbial community to the local environment [20-22,28-32].

In this study, we generated nine treatment groups using various combinations of diets and coprophagy (electronic supplementary material, table S1) to obtain parental faeces reflecting the effects of divergent gut environments, including bacterial communities. When first instars hatched, they were exposed to their natal group’s faeces. Consistent with previous findings that nymphal development of insects, including the German cockroach [58], is highly dependent on protein availability within an ecologically relevant range, different treatments strongly influenced nymphal development during the parental generation. In comparison with rodent diet (moderate protein, high carbohydrate, low lipid, low fibre), which served as our standard cockroach diet, and high-protein cat diet, low-protein rabbit diet (high carbohydrate, low lipid, high fibre) significantly impeded development, adults emerged with smaller body mass, and this group experienced higher mortality (figure 1; electronic supplementary material, figure S1). Thus, both *Blattabacterium* and the gut microbiota failed to rescue the adverse effects of low-protein diets.

Considering that microbes in the non-sterile rodent diet might have obscured a negative effect of lack of coprophagy on nymphal development, we conducted an additional experiment in which we prevented gnotobiotic nymphs with axenic guts from engaging in coprophagy and raised them on sterile diet (No-coprophagy & sterilized-rodent-diet treatment) (electronic supplementary material, figure S1). Consistent with our previous results, all nymphs reached the adult stage on pace with the D-Rodent treatment (no coprophagy and non-sterile rodent diet). Therefore, our results support the emerging view that the gut microbiome is not critical for nymphal development in the German cockroach [34]. This stands in contrast with studies that inferred fitness-related functions based on metagenomic analyses [30] or noted deficits in growth and development after eliminating the gut microbiome with antibiotics [31,32]. It is important to reiterate that, while our results suggest minimal, if any, contribution of the gut microbiome to nymphal development under laboratory conditions, they offer compelling support for its role in guiding aggregation and group affiliation. Additionally, the gut microbial community might play significant roles in other physiological processes, such as immune responses, that might not affect nymphal development or be evident under the conditions of our experiments.

## Supplementary Material

Supplementary material

Supplementary material is available online at https://doi.org/10.6084/m9.figshare.c.8570620.

## Data Availability

Data are shown in Dryad [59]. Supplementary material is available online [60].
