# Supplementary material for: Group recognition in the German cockroach, a subsocial omnivore, is based on faecal odour preference that is modulated by coprophagy, diet and learning
Source: Proc Biol Sci. Author manuscript; Available in PMC 2026 Sep 17. (PMC13585144; doi:10.1098/rspb.2025.3042)
Supplement: Supplementary file 1 [file NIHMS2204646-supplement-Supplementary_material.pdf]

Supplementary material for “Group recognition in the German cockroach, a subsocial omnivore, is based on fecal odor preference that is modulated by coprophagy, diet and learning”

Ayako Wada-Katsumata, Jamora A. Hamilton, Madhavi L. Kakumanu and Coby Schal

Proceedings of the Royal Society B
DOI: 10.1098/rspb.2025.3042

This Supplementary material includes:

Figure S1. Nymphal development in nine experimental treatments.

Figure S2. Distribution of adult emergence for the Different-Diets-only and Coprophagy & Different-Diets treatment groups.

Figure S3. Dose-response curves of aggregation preferences to fecal extracts in the Different-Diets-only, Coprophagy & Different-Diets and Coprophagy & sterilized-Rodent-diet groups.

Table S1. Experimental design for evaluation of diet and coprophagy effects on development and aggregation.

Table S2. Nymphal development and adult body mass in the parental generation (For Figure 1).

Table S3. Fecal odor preference of first instars of the offspring generation. Shelters were treated with either their own colony fecal extract or a fecal extract from a different colony (For Figure 2B).

Table S4. Fecal odor preference of first instars of the offspring generation. Shelters were treated with either their own colony fecal extract or a fecal extract from a different colony (For Figure 2C).

Table S5. Fecal odor preference of first instars of the offspring generation. Shelters were treated with either their own colony fecal extract or a fecal extract from a different colony (For Figure 3).

Table S6. Fecal odor preference of first instars of the offspring generation. Shelters were treated with either their own colony fecal extract or a fecal extract of a different colony (For Figure 4).

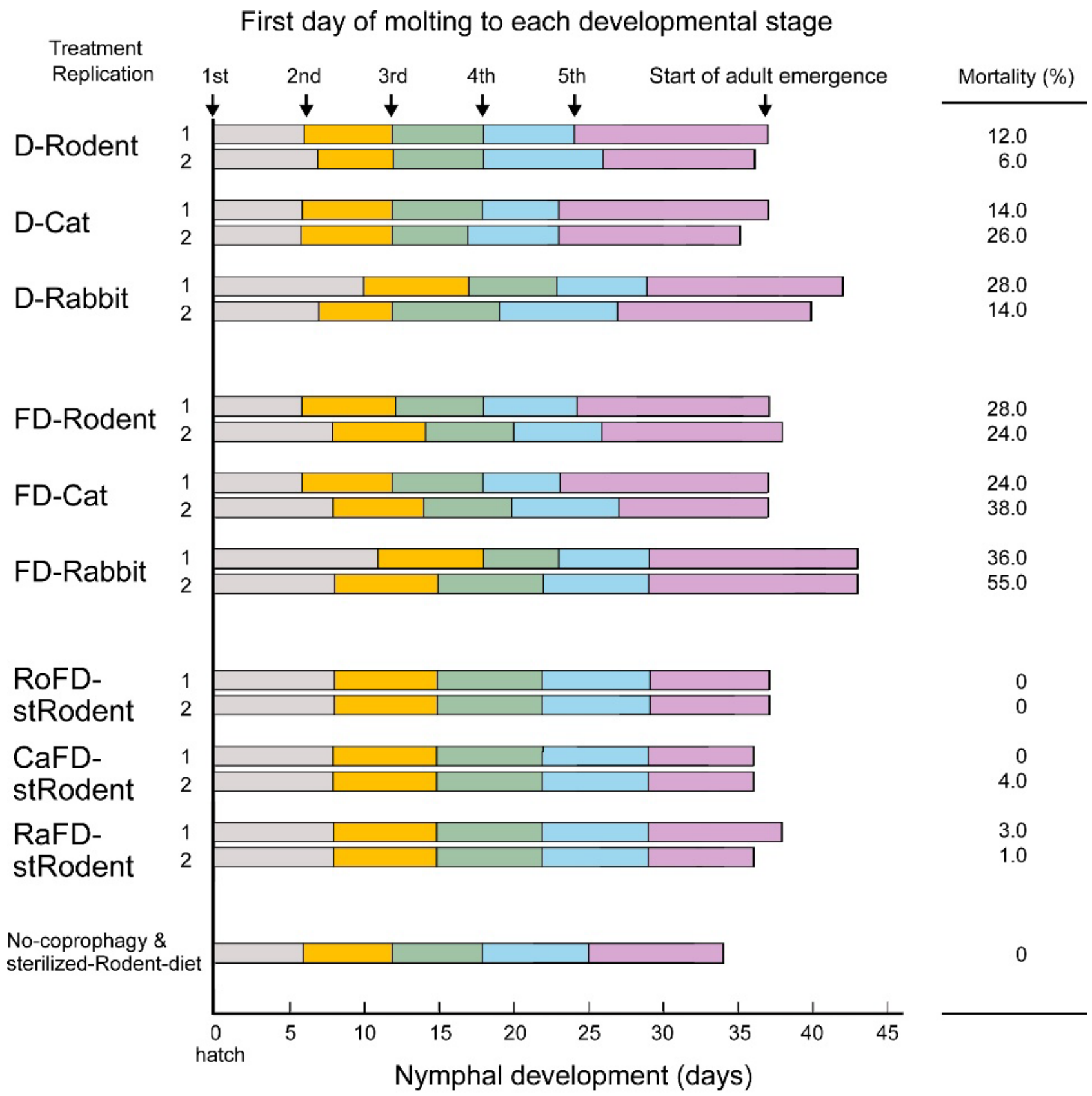

Figure S1. Nymphal development and mortality in experimental treatments (parental generation, details are shown in Table S1). Each treatment had two replications. The start of each nymphal stadium is defined by the first individual to molt to that stadium. Since there were no differences between replications, we combined the data from the two replications in each treatment. D-(diet), Different-Diets-only; F (feces) D-, Coprophagy & Different-Diets; and coprophagy on feces from groups fed different diets followed by sterile (st) rodent diet (stRodent) (Coprophagy & sterilized-Rodent-diet). We also included gnotobiotic nymphs with microbe-free guts and fed them sterilized diet (No-coprophagy & sterilized-Rodent-diet) to monitor nymphal development in the absence of a gut microbiome; 100 nymphs were included in one cage. These latter nymphs were used only for monitoring development and were not included in any data analysis.

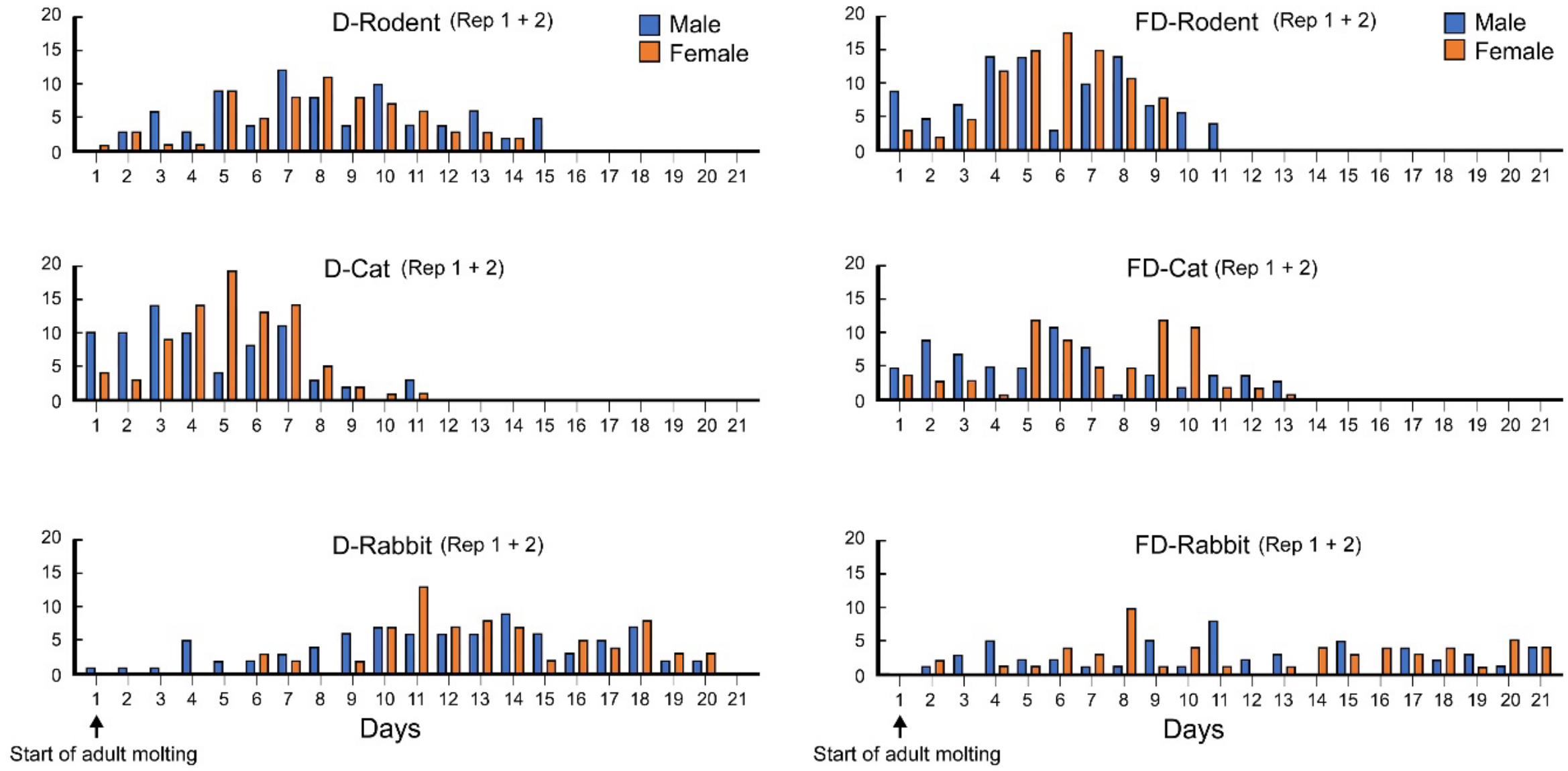

Figure S2.

Distribution of adult emergence for the Different-Diets-only and Coprophagy & Different-Diets treatment groups (parental generation, details are shown in Table S1). Day 1 indicates the first day of adult molting and it corresponds to the last day of nymphal development of the respective treatment group shown in Fig. S1.

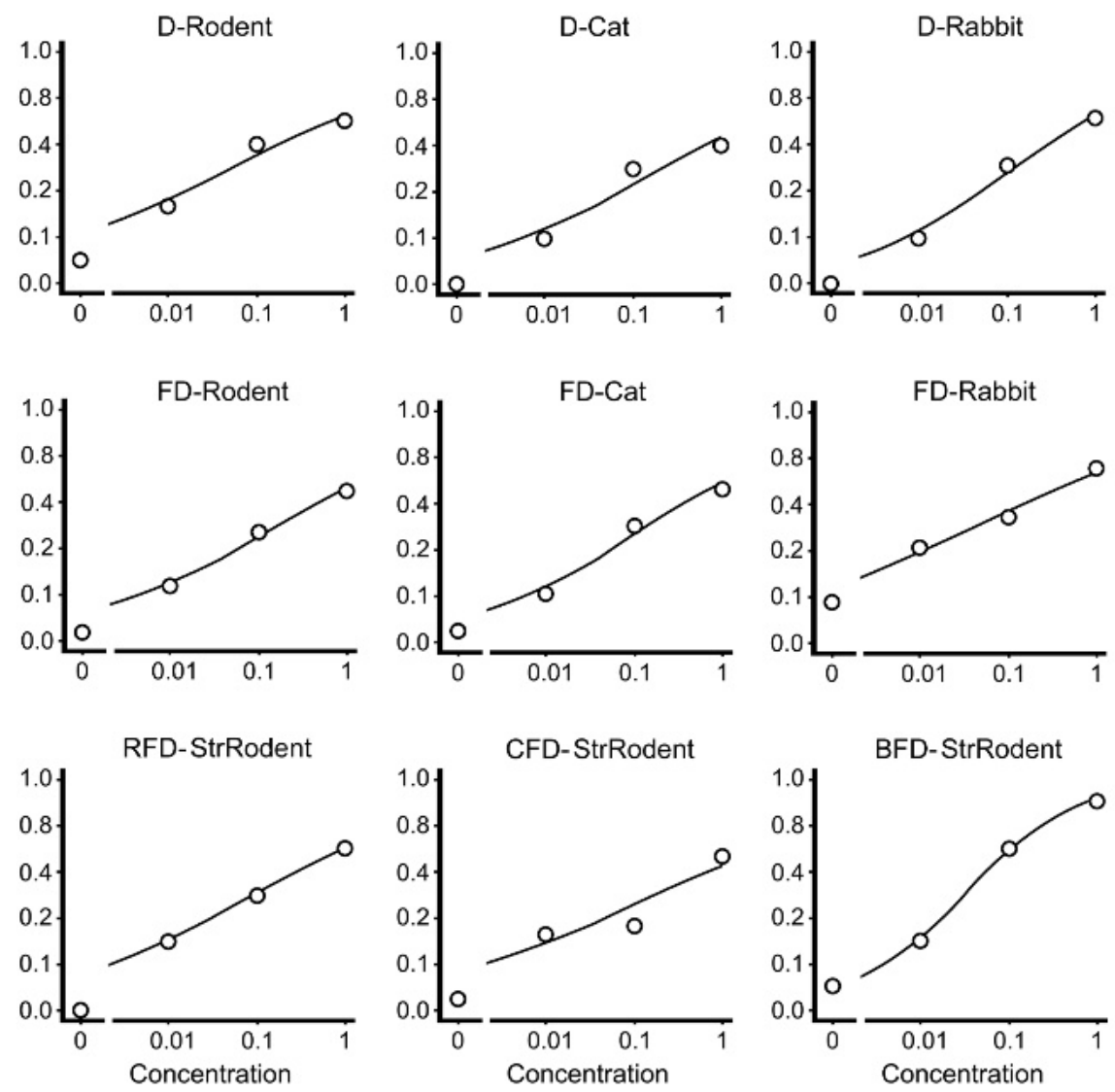

	EC_{50}	Lower	Upper	SD
D-Rodent	0.06	0.02	0.10	0.02
D-Cat	0.22	0.07	0.37	0.08
D-Rabbit	0.14	0.07	0.21	0.04
FD-Rodent	0.17	0.06	0.28	0.06
FD-Cat	0.15	0.06	0.23	0.04
FD-Rabbit	0.03	0.01	0.06	0.01
RFD-StrRodent	0.10	0.03	0.17	0.03
RCD-StrRodent	0.19	0.04	0.34	0.08
RBD-StrRodent	0.03	0.02	0.05	0.01

	EC_{70}	Lower	Upper	SD
D-Rodent	0.69	-0.04	1.42	0.37
D-Cat	2.12	-0.44	4.69	1.31
D-Rabbit	0.82	0.18	1.45	0.32
FD-Rodent	1.59	-0.18	3.35	0.90
FD-Cat	1.09	0.08	2.10	0.52
FD-Rabbit	0.49	-0.02	1.01	0.26
RFD-StrRodent	1.00	-0.05	2.05	0.53
RCD-StrRodent	3.03	-1.82	7.88	2.47
RBD-StrRodent	0.11	0.06	0.17	0.03

Figure S3 .

Dose-response curves of aggregation preferences to fecal extracts in the Different-Diets-only, Coprophagy & Different-Diets and Coprophagy & sterilized-Rodent-diet groups (offspring generation, details are shown in Table S1). First instars were given a choice between two shelters, one treated with sterile water and the other treated with a fecal extract from their own parental generation at three different concentrations: 1, 0.1 and 0.01 represent the water extracts of 50, 5 and 0.5 µg feces-equivalents/µl. Each shelter was treated with 1 µl of test solution. The effective concentrations (EC_{50} and EC_{70}) were estimated from probit analysis of the dose-response data. Based on these results, we used 15 µg of fecal extract (3 µl of 5 µg feces-equivalents/µl) in all subsequent bioassays.

Table S1. Experimental design for evaluation of diet and coprophagy effects on development and aggregation

a. Nymphal development in the parental generation

Experimental groups	Treatments	First instar nymphs [a]	Coprophagy (3 days) [b]	Diets
Different-Diets-only	D-Rodent	Gnotobiotic (axenic gut)	No [c]	Non-sterile Rodent
	D-Cat	Gnotobiotic (axenic gut)	No [c]	Non-sterile Cat
	D-Rabbit	Gnotobiotic (axenic gut)	No [c]	Non-sterile Rabbit
Coprophagy-Different-Diets	FD-Rodent	Gnotobiotic (axenic gut)	Lab colony Rodent diet feces	Non-sterile Rodent
	FD-Cat	Gnotobiotic (axenic gut)	Lab colony Rodent diet feces	Non-sterile Cat
	FD-Rabbit	Gnotobiotic (axenic gut)	Lab colony Rodent diet feces	Non-sterile Rabbit
Coprophagy-sterilized-Rodent-diet	RFD-Rodent	Gnotobiotic (axenic gut)	FD-Rodent feces	Sterile Rodent
	RFD-Cat	Gnotobiotic (axenic gut)	FD-Cat feces	Sterile Rodent
	RFD-Rabbit	Gnotobiotic (axenic gut)	FD-Rabbit feces	Sterile Rodent
Coprophagy-sterilized-Rodent-diet [c]		Gnotobiotic (axenic gut)	No [d]	Sterile Rodent

b. Aggregation bioassay in the second generation

Experimental groups	Treatments	First instar nymphs [e]	Coprophagy (3 days) [b]
Different-Diets-only	D-Rodent	Non-axenic gut, second generation	D-Rodent feces
	D-Cat	Non-axenic gut, second generation	D-Cat feces
	D-Rabbit	Non-axenic gut, second generation	D-Rabbit feces
Coprophagy-Different-Diets	FD-Rodent	Non-axenic gut, second generation	FD-Rodent feces
	FD-Cat	Non-axenic gut, second generation	FD-Rodent feces
	FD-Rabbit	Non-axenic gut, second generation	FD-Rodent feces
Coprophagy-sterilized-Rodent-Diet	RFD-Rodent	Non-axenic gut, second generation	RFD-Rodent feces
	RFD-Cat	Non-axenic gut, second generation	RFD-Cat feces
	RFD-Rabbit	Non-axenic gut, second generation	RFD-Rabbit feces

[a] Nymphs hatched from surface-sterilized oothecae from lab colony gravid females.

[b] Nymphs were kept in the hatching cage with females and fecal materials for 3 days.

[c] Extra treatment for monitoring nymphal development of gnotobiotic nymphs. One-day after hatching, nymphs received sterile Rodent diet.

[d] One-day-old nymphs started receiving non-sterile diets.

[e] Nymphs hatched in each treatment cage from oothecae of the respective parental generation females. They were exposed to feces of their parents until use in bioassay.

Table S2. Nymphal development and adult body mass in the parental generation (For Figure 1).

a. Nymphal development in the parental generation (Least Square Mean ± SE, day)			
Experimental groups	Treatments	Male	Female
Different-Diets-only	D-Rodent	40.6 ± 0.37	40.9 ± 0.30
	D-Cat	39.4 ± 0.41	40.4 ± 0.31
	D-Rabbit	46.7 ± 0.39	47.7 ± 0.33
Coprophagy-Different-Diets	FD-Rodent	43.2 ± 0.40	43.1 ± 0.35
	FD-Cat	41.3 ± 0.43	42.0 ± 0.34
	FD-Rabbit	46.8 ± 0.49	47.7 ± 0.38
Two-way ANOVA (α = 0.05)	Coprophagy	$F = 17.9$, $p < 0.0001$	$F = 21.3$, $p < 0.0001$
	Diet	$F = 121.1$, $p < 0.0001$	$F = 207.6$, $p < 0.0001$
	Coprophagy * Diet	$F = 4.7$, $p = 0.0093$	$F = 4.8$, $p = 0.0084$

b. Adult body mass (Least Square Mean ± SE, mg)			
Experiment groups	Treatments	Male	Female
Different-Diets-only	D-Rodent	27.9 ± 0.49	74.9 ± 0.88
	D-Cat	49.2 ± 0.54	77.2 ± 0.90
	D-Rabbit	44.1 ± 0.52	69.8 ± 0.97
Coprophagy-Different-Diets	FD-Rodent	48.5 ± 0.53	75.0 ± 1.01
	FD-Cat	49.6 ± 0.58	77.9 ± 0.99
	FD-Rabbit	46.1 ± 0.65	70.9 ± 1.12
Two-way ANOVA (α = 0.05)	Coprophagy	$F = 4.54$, $p = 0.034$	$F = 0.60$, $p = 0.44$
	Diet	$F = 29.42$, $p < 0.0001$	$F = 26.48$, $p < 0.0001$
	Coprophagy * Diet	$F = 1.17$, $p = 0.312$	$F = 0.13$, $p = 0.88$

Table S3. Fecal odor preference of first instars of the offspring generation. Shelters were treated with either their own colony fecal extract or a fecal extract from a different colony (For Figure 2B).

Tested nymphs of treatment	Fecal extracts	*n*	Chi-square test
	D-Rodent vs Water	40	$\chi^2(1) = 17.86$, $p < 0.0001$
D-Rodent	D-Rodent vs D-Cat	40	$\chi^2(1) = 5.76$, $p = 0.0164$
	D-Rodent vs D-Rabbit	40	$\chi^2(1) = 4.83$, $p < 0.0001$
	D-Cat vs Water	50	$\chi^2(1) = 14.08$, $p = 0.0002$
D-Cat	D-Cat vs D-Rodent	60	$\chi^2(1) = 4.57$, $p = 0.0325$
	D-Cat vs D-Rabbit	60	$\chi^2(1) = 8.34$, $p = 0.0039$
	D-Rabbit vs Water	40	$\chi^2(1) = 15.11$, $p = 0.0001$
D-Rabbit	D-Rabbit vs D-Rodent	40	$\chi^2(1) = 5.44$, $p = 0.0196$
	D-Rabbit vs D-Cat	40	$\chi^2(1) = 14.24$, $p = 0.0001$
	FD-Rodent vs Water	70	$\chi^2(1) = 33.99$, $p < 0.0001$
FD-Rodent	FD-Rodent vs FD-Cat	60	$\chi^2(1) = 35.53$, $p < 0.0001$
	FD-Rodent vs FD-Rabbit	56	$\chi^2(1) = 27.94$, $p < 0.0001$
	FD-Cat vs Water	79	$\chi^2(1) = 37.39$, $p < 0.0001$
FD-Cat	FD-Cat vs FD-Rodent	80	$\chi^2(1) = 18.51$, $p < 0.0001$
	FD-Cat vs FD-Rabbit	76	$\chi^2(1) = 14.92$, $p = 0.0001$
	FD-Rabbit vs Water	62	$\chi^2(1) = 32.27$, $p < 0.0001$
FD-Rabbit	FD-Rabbit vs FD-Rodent	80	$\chi^2(1) = 12.16$, $p = 0.0004$
	FD-Rabbit vs FD-Cat	80	$\chi^2(1) = 23.84$, $p < 0.0001$

Table S4. Fecal odor preference of first instars of the offspring generation. Shelters were treated with either their own colony fecal extract or a fecal extract from a different colony (For Figure 2C).

Tested nymphs of treatment	Fecal extracts	*n*	Chi-square test
D-Rodent	D-Rodent vs FD-Rodent	50	$\chi^2(1) = 2.08$, $p = 0.15$
FD-Rodent	D-Rodent vs FD-Rodent	30	$\chi^2(1) = 0.31$, $p = 0.58$
D-Cat	D-Cat vs FD-Cat	30	$\chi^2(1) = 1.69$, $p = 0.19$
FD-Cat	D-Cat vs FD-Cat	50	$\chi^2(1) = 0.31$, $p = 0.58$
D-Rabbit	D-Rabbit vs FD-Rabbit	30	$\chi^2(1) = 0.08$, $p = 0.77$
FD-Rabbit	D-Rabbit vs FD-Rabbit	50	$\chi^2(1) = 0.78$, $p = 0.38$

Table S5. Fecal odor preference of first instars of the offspring generation. Shelters were treated with either their own colony fecal extract or a fecal extract from a different colony (For Figure 3).

Tested nymphs of treatment	Fecal extracts	*n*	Chi-square test
RoFD-stRodent	RoFD-stRodent vs Water	60	$\chi^2(1) = 27.66$, $p < 0.0001$
	RoFD-stRodent vs CaFD-stRodent	60	$\chi^2(1) = 0.017$, $p = 0.8964$
	RoFD-stRodent vs RaFD-stRodent	60	$\chi^2(1) = 0.067$, $p = 0.7963$
CaFD-stRodent	CaFD-stRodent vs Water	75	$\chi^2(1) = 32.01$, $p < 0.0001$
	CaFD-stRodent vs RoFD-stRodent	75	$\chi^2(1) = 0.054$, $p = 0.8162$
	CaFD-stRodent vs RaFD-stRodent	60	$\chi^2(1) = 0.286$, $p = 0.5930$
RaFD-stRodent	RaFD-stRodent vs Water	75	$\chi^2(1) = 31.11$, $p < 0.0001$
	RaFD-stRodent vs CaFD-stRodent	75	$\chi^2(1) = 2.800$, $p = 0.0943$
	RaFD-stRodent vs RoFD-stRodent	75	$\chi^2(1) = 0.222$, $p = 0.6374$

Table S6. Fecal odor preference of first instars of the offspring generation. Shelters were treated with either their own colony fecal extract or a fecal extract of a different colony (For Figure 4).

Tested nymphs of treatment (1 day conditioning)	Fecal extracts	*n*	Chi-square test
N (No odor and No food)	FD-Rodent vs Water	30	$\chi^2(1) = 9$, $p = 0.027$
	FD-Cat vs Water	30	$\chi^2(1) = 8.33$, $p = 0.004$
	FD-Rabbit vs Water	30	$\chi^2(1) = 9$, $p = 0.003$
	FD-Rodent vs FD-Cat	30	$\chi^2(1) = 0.14$, $p = 0.71$
	FD-Rodent vs FD-Rabbit	30	$\chi^2(1) = 0.04$, $p = 0.85$
F (Feces)	FD-Rodent vs FD-Cat	30	$\chi^2(1) = 14.3$, $p = 0.0002$
	FD-Rodent vs FD-Rabbit	30	$\chi^2(1) = 12.5$, $p = 0.0004$
Glu (Glucose)	FD-Rodent vs FD-Cat	30	$\chi^2(1) = 0.04$, $p = 0.85$
	FD-Rodent vs FD-Rabbit	30	$\chi^2(1) = 0$, $p = 1$
FO (Fecal odor)	FD-Rodent vs FD-Cat	30	$\chi^2(1) = 0.14$, $p = 0.71$
	FD-Rodent vs FD-Rabbit	30	$\chi^2(1) = 0.33$, $p = 0.56$
Glu+FO	FD-Rodent vs FD-Cat	30	$\chi^2(1) = 8.33$, $p =0.004$
	FD-Rodent vs FD-Rabbit	30	$\chi^2(1) = 9.14$, $p = 0.002$

Tested nymphs of treatment (3 days conditioning)	Fecal extracts	*n*	Chi-square test
N (No odor and No food)	FD-Rodent vs Water	30	$\chi^2(1) = 18.2$, $p = 0.00002$
	FD-Cat vs Water	30	$\chi^2(1) = 18.2$, $p = 0.00002$
	FD-Rabbit vs Water	30	$\chi^2(1) = 21.6$, $p = 0.000003$
	FD-Rodent vs FD-Cat	30	$\chi^2(1) = 0$, $p = 1$
	FD-Rodent vs FD-Rabbit	30	$\chi^2(1) = 0$, $p = 1$
F (Feces)	FD-Rodent vs FD-Cat	30	$\chi^2(1) = 10.8$, $p = 0.001$
	FD-Rodent vs FD-Rabbit	30	$\chi^2(1) = 13.33$, $p =0.0003$
Glu (Glucose agar)	FD-Rodent vs FD-Cat	30	$\chi^2(1) = 0.17$, $p =0.68$
	FD-Rodent vs FD-Rabbit	30	$\chi^2(1) = 0.18$, $p =0.67$
FO (Fecal odor)	FD-Rodent vs FD-Cat	30	$\chi^2(1) = 0.15$, $p = 0.69$
	FD-Rodent vs FD-Rabbit	30	$\chi^2(1) = 3.20$, $p = 0.073$
Glu+FO	FD-Rodent vs FD-Cat	30	$\chi^2(1) = 5.14$, $p =0.023$
	FD-Rodent vs FD-Rabbit	30	$\chi^2(1) = 4.84$, $p = 0.028$
